# The Role of Cell Cycle Regulators in Cell Survival—Dual Functions of Cyclin-Dependent Kinase 20 and p21^Cip1/Waf1^

**DOI:** 10.3390/ijms21228504

**Published:** 2020-11-12

**Authors:** Lo Lai, Ga Yoon Shin, Hongyu Qiu

**Affiliations:** Center for Molecular and Translational Medicine, Institute of Biomedical Science, Georgia State University, Atlanta, GA 30303, USA; gshin3@gsu.edu (G.Y.S.); hqiu@gsu.edu (H.Q.)

**Keywords:** Cell cycle, CDK20, p21^Cip1/Waf1^, cancer, cardiovascular disease

## Abstract

The mammalian cell cycle is important in controlling normal cell proliferation and the development of various diseases. Cell cycle checkpoints are well regulated by both activators and inhibitors to avoid cell growth disorder and cancerogenesis. Cyclin dependent kinase 20 (CDK20) and p21^Cip1/Waf1^ are widely recognized as key regulators of cell cycle checkpoints controlling cell proliferation/growth and involving in developing multiple cancers. Emerging evidence demonstrates that these two cell cycle regulators also play an essential role in promoting cell survival independent of the cell cycle, particularly in those cells with a limited capability of proliferation, such as cardiomyocytes. These findings bring new insights into understanding cytoprotection in these tissues. Here, we summarize the new progress of the studies on these two molecules in regulating cell cycle/growth, and their new roles in cell survival by inhibiting various cell death mechanisms. We also outline their potential implications in cancerogenesis and protection in heart diseases. This information renews the knowledge in molecular natures and cellular functions of these regulators, leading to a better understanding of the pathogenesis of the associated diseases and the discovery of new therapeutic strategies.

## 1. Introduction

The cell cycle, also called the cell-division cycle, has long been an intriguing research topic because of its importance in normal cell proliferation and cancerogenesis. The mammalian cell cycle consists of four distinct phases: G1 (interphase), S (synthesis), G2 (interphase) and M phase (mitosis and cytokinesis), and a state of quiescence called G0 (inactive) phase. The activation of each phase depends on the proper progression and completion of the previous one. Both activators and inhibitors regulate the cell cycle, and the function of these regulators is crucial for normal cell growth [1,2,3].

**Figure 1:**
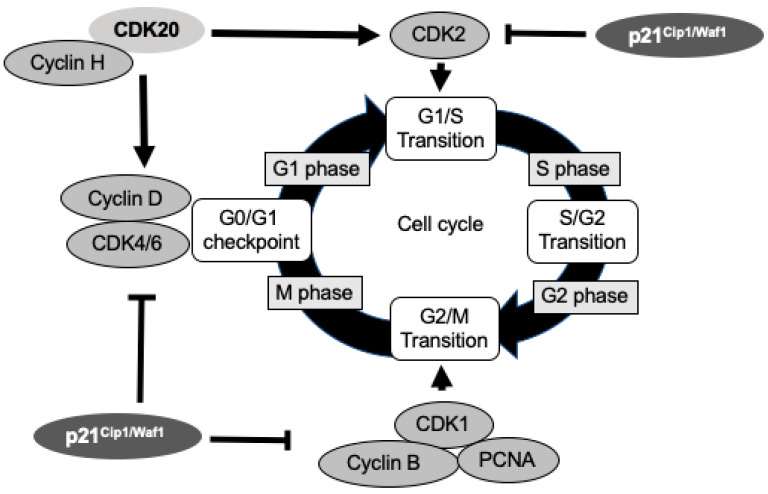
The function of cyclin-dependent kinase (CDK) 20 (CDK20) and p21^Cip1/Waf1^ in cell cycle regulation. CDK20 plays a positive regulation on cell cycles by activating CDK2 and cyclin D, while p21^Cip1/Waf1^ acts as a cell cycle inhibitor to arrest cell cycle by binding to CDK2, CDK4/6, CDK1, cyclins, and proliferating cell nuclear antigen (PCNA).

Cyclin-dependent kinase (CDK) family is a significant regulator of the cell cycle by controlling multiple checkpoints, such as: CDK2 at the G1/S and S/G2 checkpoint, CDK4 and CDK6 during G1, and CDK1 at the G1/S, S/G2 and G2/M checkpoint [4,5]. CDKs are positively regulated by CDK-activating kinases (CAKs) and are negatively regulated by CDK-inhibitors (CKIs). Among the CAKs, CDK20, also previously known as cell cycle-related kinase (CCRK) [6], has been reported to have CAK activity for CDK2 to promote the transition from G1 to S phase. It is also found to regulate the G0/G1 phase through regulating the expression of cyclin D1, a partner protein of CDK4/6 in some cancer cells [7]. In contrast, p21^Cip1/Waf1^ is one of the CKIs that is primarily associated with the inhibition of CDK2. Increasing evidence reveals that p21^Cip1/Waf1^ is also capable of inhibiting other CDK/cyclin complexes, such as CDK1 and CDK4/6 complexes [8], and thus functions as a negative regulator controlling cell cycle progression at G1 and S phase [9] (Figure 1). 

Although it is a long-held concept that CDK20 and p21^Cip1/Waf1^ are significant regulators of cell cycles, recent studies have revealed that these cell-cycle regulators also contribute to cell survival through various mechanisms inhibiting cell death processes, including apoptosis, necrosis, and autophagy. For example, p21^Cip1/Waf1^ is a significant target of p53 in response to stress stimulation, regulating various biological processes related to cell death [10,11,12,13]. An elevation of p21^Cip1/Waf1^ in certain cancer types acts as an oncogene by inhibiting apoptosis [8,14,15]. Furthermore, emerging evidence has shown that some of the CDK20 variants play distinct roles in regulating cellular functions, such as anti-apoptosis rather than cell cycle control [16], particularly in those cells with a limited capability of cell division and proliferation such as cardiomyocytes and neural cells. These findings indicate a new concept that the cell cycle regulators, such as CDK20 and p21^Cip1/Waf1^, have dual functions that may depend on the cell types. 

This review summarizes the new progress in studies of CDK20 and p21^Cip1/Waf1^ in cell growth, cell survival, and their potential implications, focusing on cancers and heart disease. The presented information redefines pre-existing knowledge about the cell cycle regulator and will open a new avenue for understanding the molecular mechanism in diseases other than cancers. 

## 2. CDK20 and p21^Cip1/Waf1^ in Cell Cycle and Cell Growth

### 2.1. CDK20 and p21^Cip1/Waf1^ Regulate Cell Cycle via CDK2

CDK2 is a member of serine/threonine (Ser/Thr) protein kinases whose activity is restricted to the G1/S phase of the cell cycle, where cells make proteins necessary for mitosis and replicate their DNA. The activation of this kinase relies on the binding of its partner cyclin protein (either A or E) to constitute a regulatory complex and the phosphorylation by CAK. For example, cyclin E binding G1 phase CDK2 is required to transition from G1 to S phase while binding with cyclin A is required to progress through the S phase [17]. The interactions induce a conformational change, allowing the Thr-160 residue of CDK2 to be exposed and phosphorylated. While cell-culture based experiments demonstrate cell cycle arrest at the G1/S transition resulting from the deletion of CDK2 [18,19], this role has been recently questioned since other reports have found that cells lacking CDK2 can still pass this transition [20]. Nevertheless, numerous evidence indicates that CDK2 is critical to the abnormal growth processes of cancer cells [21]. 

CDK20 is a newly identified small CAK protein that was first reported in HeLa cells by Kaldis and Solomon in 2000 [22]. They found that CDK20 contains all 11 conserved subdomains characteristic of Ser/Thr protein kinase and has sequence homology to both Cak1p and CDK7 groups of CAKs. CDK20 has a 43% sequence identity with CDK7, and is distinct in size from CDK7; the former peaks at 42 kDa, whereas the latter peaks at 140 kDa. It also has a substrate specificity that is different from CDK7, for example, in addition to being an activator of CDK2 as CDK7, CDK20 also favors MAK-related kinase/intestinal cell kinase (MRK/ICK) as the substrates in driving the progress of the cell cycle, which is not found in CDK7 [22]. Later on, Liu et al. further confirmed the CAK activity of CDK20 in regulating cell growth in HeLa cells. They showed that the RNAi-mediated ablation of CDK20 inhibited cell proliferation, through cell cycle G1 phase arrest by decreasing pCDK2 levels, and inhibiting CDK2 kinase activity [23]. Similar results were observed in human glioblastoma, LoVo, and DLD1 human colorectal cancer cell lines [7]. Although the accurate regulatory mechanism of CDK20 in activating CDK2 remains incomplete, merging studies have indicated the direct role of this kinase as a catalyst for CDK2 activity. For example, it has been shown that excess CDK20 phosphorylates the CDK2 on Thr-160, subsequently promoting the transition from the G1 to S phase through the phosphorylation of key target proteins, including the pRb as well as the Rb family members p130 and p107. It also releases transcription factors such as E2F that are complexed with pRb and activate the promoters of genes important in DNA synthesis [24]. Phosphorylation on CDK2 by CDK20 is stimulated by the association of CDK2 with its relevant cyclin.

On the other hand, p21^Cip1/Waf1^, also known as cyclin-dependent kinase inhibitor 1 or CDK-interacting protein 1, plays an opposite effect of CDK20 by inhibiting CDK2 activity, and thus functions as a negative regulator of cell cycle progression at G1 and S phase. p21^Cip1/Waf1^ belongs to a family of CDK interacting protein/Kinase inhibitory protein (CIP/KIP) inhibitor family with p27, p57 [25]. Those inhibitors share a homologous N-terminal domain, which contains a cyclin-binding motif 1(Cy1). This motif is indispensable for the inhibition property of p21^Cip1/Waf1,^ which allows p21^Cip1/Waf1^ binding to CDK in a region that blocks its ability to complex with cyclins, and thus prevents CDK activation [26,27]. Besides a direct interaction with the CDK2 complex, the Cy1 motif in p21^Cip1/Waf1^ mediates cell cycle arrest also through the completion with other cell cycle regulators. For example, cell division cycle 25A (cdc25A) is a phosphatase that activates the CDK2/cyclin E complex to go through the G1-S transition. Cdc25A and p21^Cip1/Waf1^ share a similar Cy1 motif and compete for interacting with the CDK2 complex, resulting in cell cycle arrest at the G1 phase [28].

### 2.2. The Role and Mechanisms of CDK20 and p21^Cip1/Waf1^ in Cell Growth

Studies have shown that both CDK20 and p21^Cip1/Waf1^ play important roles in cell growth, such as cell proliferation, division, and hypertrophy via variant mechanisms [2,11,16,25,29,30,31]. 

CDK20 has been found expressed in various human tissues, predominantly in the brain and kidney, and to a lesser extent in the liver, heart, and placenta [23]; it is also widely expressed in cell lines originating from a variety of tumor tissues. CDK20 has been indicated to play an important role in cell growth during normal tissue development. For example, it has been reported that CDK20 participates in the regulation of ciliogenesis, a process of outgrowth of cilia on the eukaryote cell surface, which is a crucial step in mammalian embryogenesis and neuron patterning by modulating the Hedgehog signaling pathway [32]. A study in *C. elegans* also indicated the essential function of CDK20 in controlling microtubule dynamics in multiple sensory neuron types [33]. In addition, CDK20 was found involved in the pathogenesis of diseases, particularly in cancers. In glioblastoma cells, knockdown CDK20 blocks cancer cell proliferation [16,34,35]. These findings indicate that CDK20 is a key regulator of cell growth in various cell types. 

Despite the lack of full understanding of the functions and differences in cell lines, CDK20 is currently known to regulate cell growth in both a CAK and non-CAK manner in different cells, which have been reviewed previously [7]. In addition to the direct effect in phosphorylating CDK2 on Thr-160 as mentioned above in the cell cycle, CDK20 may also bind and/or phosphorylate CDK7 (or other CDKs) [36]. Furthermore, other than the activation of CDK2, reports indicate that CDK20 promotes cell growth through activating MRK/ICK. CDK20 has also been found to regulate cell growth through other mechanisms such as regulating β-catenin-T-cell factor (TCF) signaling, Wingless-related integration site (Wnt) signaling pathway, phosphorylation of glycogen synthase kinase 3β (GSK3β) at Thr390, or inducing an expression of cyclin D1 [7]. However, these potential mechanisms still need further confirmation. 

p21^Cip1/Waf1^ is also found to be widely involved in cell growth through different mechanisms. One of the mechanisms is mediated by its inhibitory effect on CDKs. Although it is primarily associated with the inhibition of CDK2, studies show that p21^Cip1/Waf1^ is capable of inhibiting almost all cyclin /CDK complexes [37]. For example, the low concentration p21^Cip1/Waf1^ promotes proliferation through the assembly and activation of cyclin D/CDK4/6 complexes [38,39], and phospho-p21^Cip1/Waf1^ promotes cyclin B/CDK1 complexes activities [15]. In addition, Insinga et al. showed that irradiation of hematopoietic stem cells results in the upregulation of p21^Cip1/Waf1^, causing p53 inhibition, ultimately leading to cell cycle entry and symmetric self-renewing divisions [40]. Furthermore, p21^Cip1/Waf1^ can regulate cell proliferation by interacting with the PCNA, a DNA polymerase accessory factor, which plays a regulatory role in the S phase. PCNA is a regulator of DNA synthesis, and its expression is controlled by E2F (E2 factor) transcriptional factor containing complexes [27]. Interaction between p21^Cip1/Waf1^ and PCNA ensures the progress of the cell cycle. p21^Cip1/Waf1^ displaces PCNA partners to control DNA replication in the S phase. In return, PCNA also controls the expression of p21^Cip1/Waf1^ in the S phase to prevent the upregulation of p21^Cip1/Waf1^, which arrests the cell cycle [27]. Besides, DNA damage causes p21^Cip1/Waf1^ to bind CDK1 complexes, which inhibits the catalytic function of the complex, subsequently bringing about cell cycle arrest [41,42]. Moreover, p21^Cip1/Waf1^ plays a dual role in the process of cell hypertrophy, a process by which a cell increases its size, either in physiological or maladaptive conditions. Studies have shown that the absence of p21^Cip1/Waf1^ did not affect transforming growth factor (TGF)-beta’s action on proliferation, but did decrease TGF-beta-induced hypertrophy in p21^Cip1/Waf1^ knockout mesangial cells compared with the control. In the same study, it was discovered that the expression of p21^Cip1/Waf1^ was required for the initial phase of hypertrophy, and its absence caused a significant delay in hypertrophy [43]. These results suggest that p21^Cip1/Waf1^ promotes hypertrophy. However, other studies have provided conflicting evidence of p21^Cip1/Waf1^ in cell hypertrophy. For example, upregulating p21^Cip1/Waf1^ was found to be associated with the inhibition of cardiomyocyte hypertrophy via a cardiac-restricted protein, called CHAMP (cardiac helicase activated by MEF2 protein) during prenatal and postnatal development of the heart [44], while a downregulation of p21^Cip1/Waf1^ was found in aortic constriction-induced cardiac hypertrophy in adult hearts [9], which was supported by a recent study showing that p21^Cip1/Waf1^ protects cardiac hypertrophy [10]. Nevertheless, p21^Cip1/Waf1^ KO mice can grow normally despite exhibiting impaired G1 checkpoint control [45,46], indicating the complexity of its functions. 

## 3. CDK20 and p21^Cip1/Waf1^ in Cell Death and Survival

For the past decades, research on CDK20 and p21^Cip1/Waf1^ has been focused on their cell-cycle regulator properties. Recently, increasing evidence has revealed that these cell cycle regulators also actively participate in regulating cell death and survival pathways. Interestingly, studies indicate that the effect of CDK20 in cell survival is in an isoform-dependent manner, while p21^Cip1/Waf1^ regulates multiple cell death signaling according to its subcellular localization. 

### 3.1. CDK20 in the Regulation in Cell Survival and Its Variants

In addition to the effects in cell cycle and cell growth mentioned above, CDK20 was also involved in cell survival in some types of cancers. For example, it has been shown that knockdown of CDK20 slows down HeLa cells’ growth and complete silencing of this protein is lethal. Pharmacological inhibition of CDK20 causes both cycling and noncycling cancer cell death [7,47]. These findings further highlight the importance of the pro-survival role of CDK20 independently from its effect on the cell cycle.

A recent study has further revealed the pro-survival role of CDK20 in cardiomyocytes, which are characterized by a limited capability of proliferation. The study indicated that the CDK20 expressed in adult cardiomyocytes is a unique splice variant that is different from those in normal cells with the capability of cell division, and that it is also distinct from cancer cells. This splice variant of CDK20 was found to play a critical role in cell survival instead of the cell cycle in cardiomyocytes, since it could not activate CDK2 which is typically functioning in other cells. Overexpression of this CDK20 variant protects cardiomyocytes against stress-induced cell death instead of cell division [47,48], indicating a different role of CDK20 in the heart.

**Figure 2:**
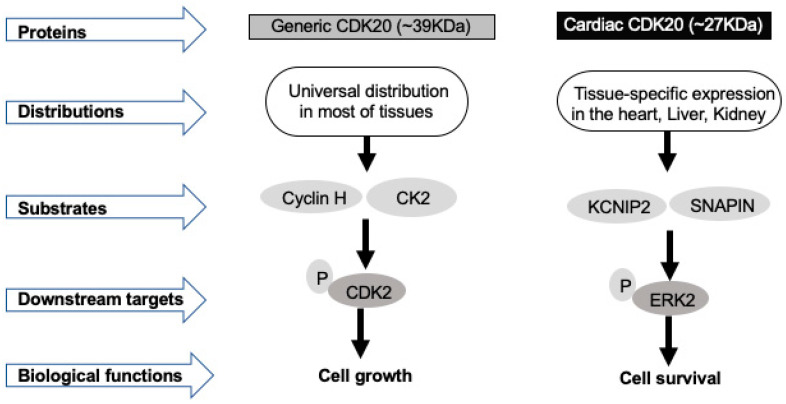
Difference of two variants of CDK20. Generic CDK20 and cardiac CDK20 are the two isoforms of CDK20 with differences in tissue distributions, substrates, downstream targets, and biological functions. While generic CDK20 widely expresses in most tissues and acts as a cell cycle regulator to promote cell growth, cardiac CDK20 expresses predominantly in the heart, liver, and kidney, and promotes the cell survival pathway.

Although the regulatory signaling pathways by which CDK20 mediates cell survival are not fully understood, it appears that this effect relies on the unique variant expressed in the specific cells. Increasing evidence reveals that CDK20 exists as a few alternative RNA splicing, and more new variants are being discovered. According to the most recent updated information from NCBI resources (Gene ID: 23552, updated on 1 June 2020), there are at least seven transcription variants in mammalian cells. Among these reported variants, two identified proteins with a molecular weight 27 KDa or 39 KDa, respectively [16,47,49], attracted the most attention because of their unique tissue and cellular distributions and their distinct functions as well as their close link with the development of cancers and heart diseases. As shown in Figure 2, these two isoforms differ in tissue distribution, substrates, and protein interaction. While the "generic" isoform can be found in most tissues, the smaller variant can only be found in some specific tissues, such as the heart, liver, and kidneys [47]. 

As this smaller variant was first identified in the heart, it was called a “cardiac CDK20” [47]. The generic CDK20, but not the cardiac variant, interacts with cyclin H and casein kinase 2 (CK2) and phosphorylates CDK2. Reciprocally, the cardiac variant has been reported to interact with voltage-gated potassium (Kv) channel interacting protein 2 (KCNIP2), SNAP-associated protein (SNAPIN), and Paladin ATP-binding cassette, and is involved in the activation of extracellular signal-regulated kinase (ERK) survival signaling [47,48]. There is also a difference in the expression between these two isoforms of CDK20 during the heart’s development. While the expression of the generic CDK20 is predominant in the neonatal heart, it decreases markedly after one month of the birth, as cardiac myocytes mature. Remarkably, the cardiac isoform shows a reciprocal pattern of expression, i.e., a deficient level of expression in the neonatal tissue and predominant expression in the mature heart [47]. This shift of the expression between these isoforms may be associated with the underlying mechanism of the loss of cell proliferation capability in adult cardiomyocytes.

### 3.2. The Dual Roles of p21^Cip1/Waf1^ in Cell Death and Survival and Its Compartment-Specific Effects

**Figure 3:**
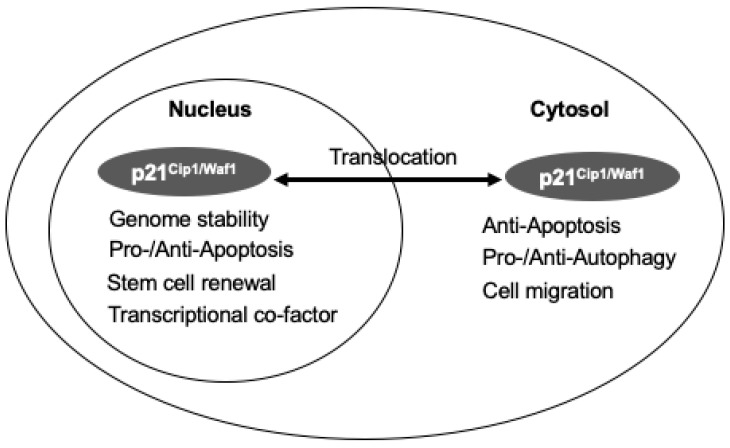
The dual function of p21^Cip1/Waf1^ depends on the subcellular distribution. Functions of p21^Cip1/Waf1^ are different in the nucleus and cytosol localization. The nucleus p21^Cip1/Waf1^ maintains genome stability, regulates apoptosis, involves stem cell renewal, and acts as a transcriptional co-factor, while the cytosol p21^Cip1/Waf1^ binds to caspase to inhibit apoptosis and regulate autophagy.

p21^CIP1/WAF1^ is also involved in determining cell death, including apoptosis, necrosis, and autophagy. It has been shown that the intercellular localization and phosphorylation of p21^Cip1/Waf1^ affect its function. While nuclear p21^Cip1/Waf1^ inhibits CDKs, consequently inhibiting cell cycle progression by halting the cell cycle at checkpoints, cytoplasmic p21^Cip1/Waf1^ prevents apoptosis by direct interference of multiple apoptotic pathways existing in the cell. For example, phosphorylation of p21^Cip1/Waf1^ inhibits interaction with CDK/cyclin complexes and PCNA in the nucleus, while it enables p21^Cip1/Waf1^ to interact with multiple proteins in the cytoplasm to prevent apoptosis. It has also been shown that the effects of p21^Cip1/Waf1^ on autophagy are compartment-specific [8,42] (Figure 3). Although the tertiary structure of p21^Cip1/Waf1^ is still unclear, researchers believe that p21^Cip1/Waf1^ transformation can be changed depending on its binding proteins. There are multiple binding sites and phosphorylation sites in p21^Cip1/Waf1^. These properties result in a long and expanding list of binding partners [8,50]. 

A normal cell requires activation of the endogenous anti-apoptotic mechanism to repair the damaged DNA caused by the stress [51]. p21^Cip1/Waf1^ is thought to be one of the mediators of this anti-apoptotic mechanism. Insinga et al. found that the irradiation of hematopoietic stem cells results in the upregulation of p21^Cip1/Waf1^, responsible for the resistance to apoptosis [40]. With a defective p21^Cip1/Waf1^ response, human colon cancer cells result in apoptosis when treated with chemotherapy drugs via activation of caspase 9, while cells with a normal p21^Cip1/Waf1^ expression showed no such activation [52]. It has been reported that p21^Cip1/Waf1^ plays an anti-apoptotic role via an increase of both p14ARF (ARF tumor suppressor) and p53 levels, and an alteration of the BCL2 Associated X (Bax)/B-cell lymphoma 2 (Bcl-2) ratio [52], inhibition downstream of caspase cascade [51,53]. The ability of p21^Cip1/Waf1^ to inhibit apoptosis in response to replication fork stress has also been reported [54]. Thus, p21^Cip1/Waf1^ interferes with apoptosis via multiple mechanisms, including transcriptional regulation, binding to pro-apoptotic cytoplasmic products, and CDK inhibition. 

In addition to the anti-apoptosis effect, p21^Cip1/Waf1^ has also been linked to the process of necrosis. A study found that animal hepatocytes with a deficient p21^Cip1/Waf1^ were resistant to pharmacologically induced necrotic injury. This suggests that p21^Cip1/Waf1^ plays an essential role in the process of necrosis. These p21^Cip1/Waf1^-deficient hepatocytes also proliferated the most. However, using caspase-3 activation as a marker, researchers discovered that apoptosis counterbalances this proliferation. It is important to distinguish that although p21^Cip1/Waf1^ functions in two seemingly opposing parts, timing is critical for determining whether p21^Cip1/Waf1^ will be anti-apoptotic or pro-necrotic. Early-onset of p21^Cip1/Waf1^ expression contributes to necrosis, while later expression during repair causes the termination of proliferation [55]. There is an increase in p21^Cip1/Waf1^ expression before tumor necrosis factor-induced necrosis. Interestingly, when p21^Cip1/Waf1^ was inhibited, the G2/M phase delay is obliterated, causing cell death to occur [56]. 

Furthermore, p21^Cip1/Waf1^ is reported to involve mechanisms of cell autophagy. For example, in human telomerase reverse transcriptase (hTERT)-immortalized sensitized fibroblast cell lines, overexpressed p21^Cip1/Waf1^ results in autophagy induction and mitochondrial dysfunction upon starvation [57]. In a study to determine whether p21^Cip1/Waf1^ affects the type of programmed cell death, C_2_-ceramide was used as a stress inducer. It was found that cells positive for p21^Cip1/Waf1^ resulted in cell apoptosis. The same treatment on cells that were negative for p21^Cip1/Waf1^ caused autophagy instead [58]. On the other hand, p21^Cip1/Waf1^ can inhibit the autophagic pathway and trigger apoptosis under lethal stress in mouse embryonic fibroblasts [13,58]. These suggest that p21^Cip1/Waf1^ has a crucial role in determining which pathway a cell will follow. This information is summarized in Figure 3.

## 4. The Relevance of CDK20 and p21^Cip1/Waf1^ in Cancers

As shown in Table 1, CDK20 and p21^Cip1/Waf1^ are involved in various cancers. CDK20 has been of particular interest in various cancer research lines because of its activating role in cell proliferation. In ovarian carcinoma, CDK20 knockdown in cells led to G1 phase cell cycle arrest, and CDK20 overexpression caused cell proliferation in vitro and tumor growth in vivo, and is positively correlated with an advanced stage of ovarian cancer. CDK20 was also demonstrated to promote ovarian carcinoma cell proliferation via regulation of cyclin D1 and is a predictor of outcome in patients with ovarian carcinoma [59]. In glioblastoma cells, CDK20 expression allows cell proliferation [34]. In this case, knockdown of CDK20 leads to G1 phase cell cycle arrest and decreased CDK2 phosphorylation, suppressing the growth of glioma cells in vivo, implicating CDK20 as an oncogene for this type of cancer. It is thought that CDK20 is also responsible for the proliferation of other cancers such as cervical carcinoma, osteosarcoma, and colorectal carcinoma [36]. The mechanism by which CDK20 regulates cancer growth is typical to increase cell growth through activation of CDK2 via the mechanisms that we described above. However, some non-CAK effects have also been observed. It has been reported that recombinant CDK20 has no CAK activity on CDK2 or CDK2 complex in vitro, and knockdown CDK20 in U2OS cells did not arrest the cell cycle [60]. Similarly, CDK20 also was found in ovarian carcinoma cell lines and CDK20 has no CAK activity on CDK2 but promotes cell proliferation via increases in cyclin D expression [59]. There are also reports indicating that CDK20 might involve cancer cell survival by other signaling such as via MRK/ICK signaling via phosphorylation of the essential Thr-157 in their T-loop in prostate cancer cells [7,61]. Block CDK20 activity causes accumulation of ICK and inhibition of cell-cycle entry [5,34,60,62,63]. CDK20 may also be a positive component in the Wnt signaling pathway, present in the cell to aberrantly activate β-catenin and to provoke tumor-associated cell proliferation [64].

In addition, studies of p21^Cip1/Waf1^ also focus on cancer cell lines by observing its effects on cell proliferation and survival. It has been found that p21^Cip1/Waf1^ plays dual roles in the tumor growth depending on the type of cancer, intracellular localization, and associated treatments [8,25]. Overexpression of p21^Cip1/Waf1^ has been found in association with prostate, ovarian, cervical, breast, and esophageal carcinomas and human gliomas [8,14,30,76]. Reciprocally, loss of p21^Cip1/Waf1^ expression is associated with carcinogenesis, along with p53 inactivation [77]. p21^Cip1/Waf1^ is considered anti-oncogenic because its deletion induces spontaneous tumor growth in mice [78]. For example, mice deficient in p21^Cip1/Waf1^ developed tumors on average at 16 months, while mice that were not deficient remained tumor-free after 24 months [78]. It was noted that p21^Cip1/Waf1^-deficient mice were protected from radiation-induced carcinogenesis through a p53-dependent cell cycle arrest mechanism [78]. However, p21^Cip1/Waf1^ can also be considered oncogenic, as localized cytoplasmic p21^Cip1/Waf1^ inhibits caspase activity [25,42]. Furthermore, p21^Cip1/Waf1^ is responsible for acting as a shield for tumor cells against DNA damage cytotoxic effects [79]. Upon DNA damage, such as by irradiation, p21^Cip1/Waf1^ regulates p53 stability, subcellular localization, and activity in a negative feedback loop through murine double minute 2 (MDM2) [52], or a positive feedback loop through ataxia-telangiectasia mutated (ATM)-p21^Cip1/Waf1^ pathway [80]. Recently, a research group using mathematical modeling suggested that DNA-damage induced Checkpoint kinase 1 (Chk1) activation regulates p21^Cip1/Waf1^/p53 balance, and the activation of Chk1 varies by amount of cell types [81]. 

## 5. Potential Role of CDK20 and p21^Cip1/Waf1^ in Cardio-Protection

Cardiomyocyte is a unique type of cell that is distinct from most other cells because of its non-regenerative nature in the adult heart. Since cardiomyocyte proliferation terminates rapidly after birth, any cause-induced cardiomyocyte death would result in an irreversible reduction of the numbers of these cells in the heart, eventually leading to heart failure. Thus, promoting cardiomyocyte survival is extremely important in protecting the heart from functional failure. 

There are very few studies on CDK20 in the heart until discovery of the unique variant of the cardiac CDK20 in the cardiomyocyte [47]. As stated, the cardiac variant of CDK20 is interesting due to the following reasons: first, it is specifically expressed in the heart; secondly, it losses the effect on activating the cell cycle; third, its expression dramatically increases in the heart after birth while generic CDK20 declines. These findings together imply that the shift of the cardiac CDK20 in adult cardiomyocytes may contribute to the limited capability of regeneration in cardiomyocytes after birth. Importantly, cardiac CDK20 has been linked to heart diseases. It has been found that CDK20 was significantly decreased in myocardial ischemic hearts in different animal models [48]. Overexpression of cardiac CDK20 protects cardiomyocytes from death and prevents cardiac stress-induced heart failure [48]. Further investigation on this novel variant of CDK20 may yield a unique target for diagnostic markers and treatment therapies for patients with ischemic cardiac diseases and heart failure. 

The prior studies have been focused on the mechanism that mediates the termination of the cell proliferation in cardiomyocytes and found that the activation of p21^Cip1/Waf1^ plays a direct part in cardiomyocyte withdrawal from the cell cycle [82]. The knockdown of p21^Cip1/Waf1^ caused neonatal and adult cardiomyocytes to re-enter the S phase [82]. There is a stark contrast between cardiomyocytes and skeletal muscle cells that involves the cell cycle. Skeletal muscle p21^Cip1/Waf1^ knockdown causes aberrant mitosis and apoptosis, commonly leading to mitotic catastrophe and cell death. However, cardiac muscle p21^Cip1/Waf1^ knockdown does not cause significant DNA damage or apoptosis but results in reactivation of DNA synthesis. This reactivation is correlated with a colossal change in the morphology of the cardiomyocytes that is suggested to be a pre-requisite for proliferation [82]. These studies indicate that p21^Cip1/Waf1^ is essential for an adult cardiomyocyte to maintain its status as a terminal proliferation.

Studies also show that p21^Cip1/Waf1^ is involved in cardiac diseases by regulating the cell survival of cardiomyocytes in the adult heart. Since p21^Cip1/Waf1^ is a dual functional protein, whether it is pro-apoptotic or anti-apoptotic depends on its cellular localization. It was found that under cardiac stress conditions, such as chronic hypoxia, acute ischemia, and hyperthermia, p21^Cip1/Waf1^ cytoplasmic localization is increased, suggesting that p21^Cip1/Waf1^ switches function from cell cycle proliferation to survival [83]. Research has shown that levels of p21^Cip1/Waf1^ revert to a human fetal heart pattern in both acute and end-stage heart failure, showing a decrease in p21^Cip1/Waf1^ with an increase in p53 [84]. These studies suggest that p21^Cip1/Waf1^ expression may be cardio-protective under extreme stress. Therapy targeting this role can perhaps benefit the patients with myocardial infarct and heart failure by stimulating cardiomyocytes to re-enter the cell cycle and proliferate, replacing damaged or lost cells in the heart. However, this type of therapy needs to be monitored carefully due to potential side effects from the multiple effects of p21^Cip1/Waf1^. Increases in p21^Cip1/Waf1^ expression in aged mouse hearts have also been reported [85]. However, the underlying mechanism remains unclear.

## 6. Clinical Potential

Studies have shown that CDK20 and p21^Cip1/Waf1^ serve as important regulators in tumorigenicity and are functionally connected to a broad range of cell signaling pathways with important functions in cell cycle progression, cell proliferation, and malignant transformation, indicating that these regulators may serve as novel prognostic markers and may be promising candidates as a molecular target for cancer therapy for some types of cancer. However, the relative clinical applications remain limited. Besides acting as a potential therapeutic cancer target, CDK20 and p21^Cip1/Waf1^ have also been considered targets of preventing chemotherapy resistance treatment in cancer. For example, in lung cancer, CDK20 was reported involved in the in radio-chemotherapy resistance by interacting with the Kelch-like ECH-associated protein 1 (KEAP1)–nuclear factor erythroid-2-related factor 2 (NRF2) pathway. CDK20 interacts with KEAP1, which is the inhibitor of NRF2, activates the survival pathway, and results in radio-chemoresistance [70,86]. Similarly, the anti-apoptotic function p21^Cip1/Waf1^ induces chemotherapy resistance in renal cell carcinoma and breast cancer [28]. Several small-molecule inhibitors of p21^Cip1/Waf1^ with potential clinical benefits prevent chemotherapy resistance in kidney cancer [87]. However, a meta-analysis report on published esophageal cancer patient data gives a controversial conclusion. They found that patients with low p21^Cip1/Waf1^ expression have a poor outcome with chemotherapy, while high expressed patients react vice versa, which could be due to the dual function of p21^Cip1/Waf1^ [88]. 

## 7. Conclusions and Future Directions

In summary, CDK20 and p21^Cip1/Waf1^ play essential roles in both the cell cycle and cell survival. Their comprehensive biological functions depend on multiple factors, including their variants, cell types, subcellular translocation, onset timing, and interacting proteins. Further investigating the underlying mechanisms would lead to a deeper understanding of relative diseases’ pathogenesis and provide potential therapeutic avenue. It is of utmost importance that the function of CDK20 and p21^Cip1/Waf1^ in the heart is elucidated because they can have major implications on the development of novel therapies for heart diseases, thus improving the quality of life and life expectancy for affected patients.

Since cell cycle regulators have been extensively studied on proliferative cells and cancer cells, their cell survival roles and the underlying mechanisms remain largely unknown. Future research could be focused on those cells with a limited capability of cell proliferation, such as cardiomyocytes and neural cells, since the regeneration of these cells is still a scientific challenge despite their importance. Understanding the effect of these cell cycle regulators inside these cells will bring new insights for discovering the therapeutic strategy for damaging these tissues and impaired function. 

Also, the role of CDK20 is different among the variants, so exploring the mechanism controlling the alternative splicing of CDK20 in other cells will help to understand the cell function and pathogenesis of various diseases. 

Furthermore, it has been shown that p21^Cip1/Waf1^ plays multiple roles in cell survival and cell growth that largely depend on the cell types and subcellular translocation. Understanding the mechanisms driving the translocations of p21^Cip1/Waf1^ in these cells will open a new avenue to control cellular function under disease conditions. 

Finally, since CDK20 and p21^Cip1/Waf1^ share many similar functions in multiple cells, further attention should be drawn to discover the links between p21^Cip1/Waf1^ and CDK20. There are a few potential common pathways between these two regulators: first, they regulate the cell cycle via acting on the CDK2. Second, it has been shown that CDK20 activates the ERK1/2 pathway, which subsequently induces p21^Cip1/Waf1^ expression [89]. Other links could involve the Wnt/beta-catenin pathway or even a direct link between CDK20 and p21^Cip1/Waf1^. CDK20 could also be possible as the upstream regulator for the translocalization of p21^Cip1/Waf1^ driving to an anti-apoptotic pathway. These studies would result in the discovery of an integrating mechanism in regulating cell growth and cell survival that could have a great application for treatment of various cell-cycle related diseases, including but not limited to cancer and heart disease, providing a significant clinical translational potential. 

## Figures and Tables

**Table 1:** Function of CDK20 and p21^Cip1/Waf1^ in cancer cell line/system.

Cancer Type	CDK20	p21^Cip1/Waf1^
Ovarian	cell proliferation [59]	cell survival [65]
Glioblastoma	cell proliferation [36]	cell proliferation [66]
Cervical carcinoma	cell proliferation [23]	High expression function unclear [67]
Osteosarcoma	cell proliferation [60]	Tumor suppressor [68]
Colorectal carcinoma	cell proliferation [60]	Tumor suppressor [69]
Lung cancer	Oncogene [70]	Tumor suppressor [71]
Head and neck cancer	N/A	Tumor suppressor [72]
Breast cancer	Oncogene [16]	Cell proliferation [73]
Liver cancer	Oncogene [74]	Tumor suppressor [75]
